# Inflammatory myofibroblastic tumor directly invading the right first rib treated with oral steroids: a case report

**DOI:** 10.1186/s12890-024-02873-6

**Published:** 2024-02-02

**Authors:** Ryo Watanabe, Satoshi Ano, Norihiro Kikuchi, Michiko Saegusa, Rie Shigemasa, Yuzuru Kondo, Nobuyuki Hizawa

**Affiliations:** 1 Department of Respiratory Medicine, National Hospital Organization Kasumigaura Medical Center, 2-7-14 Shimotakatsu, 300-8585 Tsuchiura, Ibaraki Japan; 2 Department of Respiratory Medicine, University of Tsukuba, Tsukuba, Japan; 3 Department of Diagnostic Pathology, National Hospital Organization Kasumigaura Medical Center, Tsuchiura, Japan

**Keywords:** Asthma, Anaplastic lymphoma kinase, Corticosteroid, Inflammatory myofibroblastic tumor

## Abstract

**Background:**

We present a case of an inflammatory myofibroblastic tumor cured with a short period of steroid administration, a treatment previously unreported for such cases.

**Case presentation:**

A 49-year-old man had a chief complaint of chest pain for more than 3 days. Computed tomography (CT) revealed a tumoral lesion suspected to have infiltrated into the right first rib and intercostal muscles, with changes in lung parenchymal density around the lesion. The maximal standardized uptake value on 18 F-fluorodeoxyglucose positron emission tomography/computed tomography was high (16.73), consistent with tumor presence. CT-guided biopsy revealed an inflammatory myofibroblastic tumor with no distant metastases. Surgery was indicated based on the disease course. However, he had received an oral steroid before the preoperative contrast-enhanced CT scan due to a history of bronchial asthma, and subsequent CT showed that the tumor shrank in size after administration; he has been recurrence-free for more than a year.

**Conclusions:**

Surgery is still the first choice for inflammatory myofibroblastic tumors, as the disease can metastasize and relapse; however, this condition can also be cured with a short period of steroid therapy.

## Background

An inflammatory myofibroblastic tumor (IMT) is characterized by the proliferation of myofibroblasts. IMTs are rare, accounting for only approximately 0.04% of all lung tumors and occurring mainly during childhood and adolescence. Further, there is no sex-specific difference in incidence [1]. Hematological findings include increased hemopoiesis, increased platelets, increased polyclonal γ-globulin, and elevated levels of interleukin (IL)-6 and IL-1β; however, no specific markers have been reported [2].

While the exact etiology of this disease is unknown, it is a relatively rare condition defined as a benign-malignant intermediate tumor that exhibits characteristics such as metastasis, recurrence, and marked infiltration of inflammatory cells, mainly lymphocytes and plasma cells [3, 4]. IMTs are found in the abdominopelvic region, lungs, retroperitoneum, and other organs [5]. However, reports of IMT occurring subcutaneously in the chest wall and invading the ribs are rare [6], and the present case occurred as a subcutaneous tissue tumor, not a lung tumor. Furthermore, to the best of our knowledge, there are no reported cases of IMTs that resolved only with short-term steroid treatment rather than surgical treatment. Although surgery is considered the first-line treatment, we present a case of an IMT cured with a short period of steroid administration.

## Case presentation

**Fig. 1:**
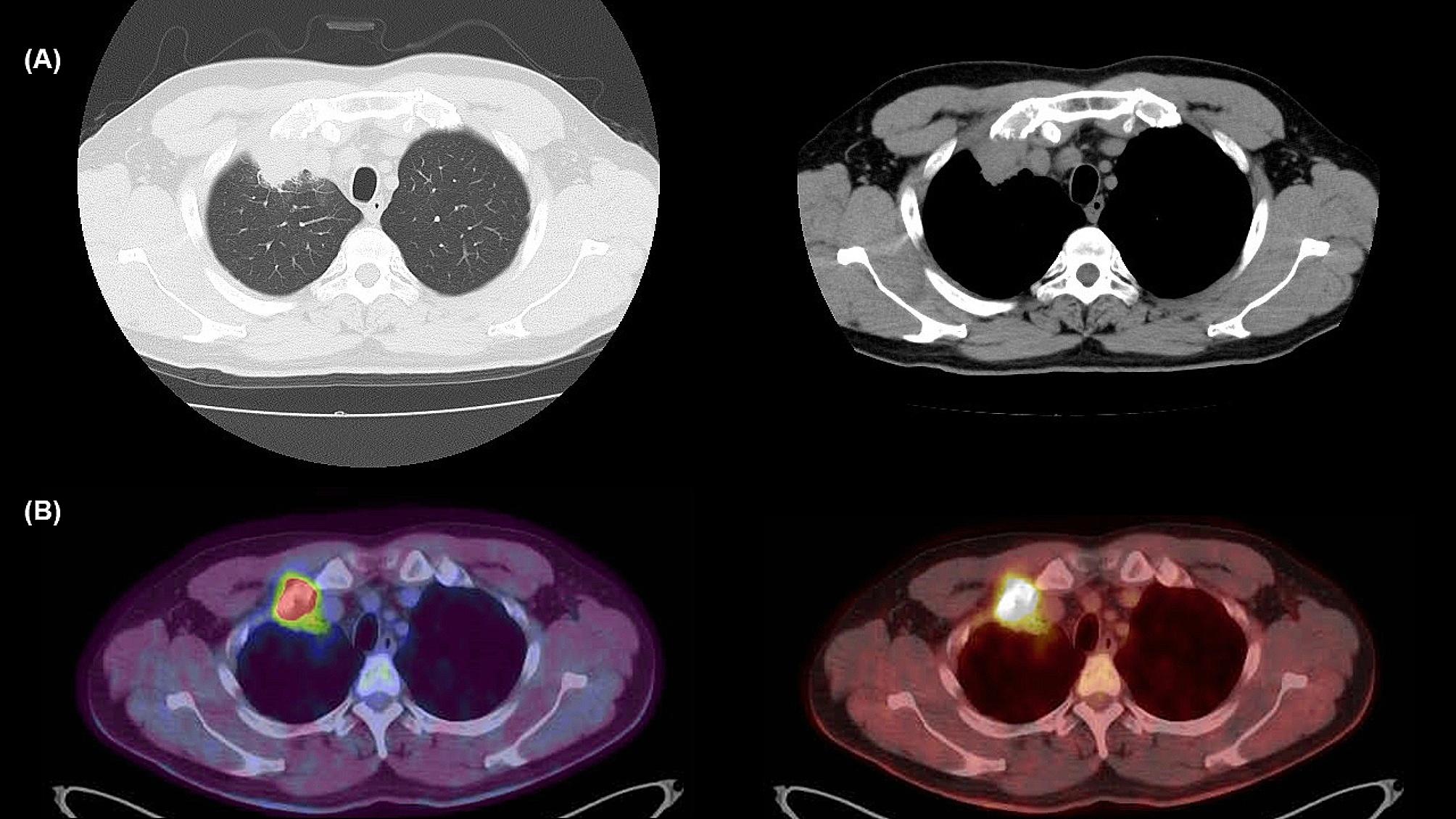
**(A)** Computed tomography on the 12th day post-admission shows a tumoral lesion suspicious of infiltration into the right first rib and intercostal muscles, with changes in the density of the lung parenchyma around the tumor. These findings are suggestive of extension of the lesion along the lymphatic vessels and stroma. **(B)** 8 F-Fluorodeoxyglucose positron emission tomography-computed tomography on the 17th day post-admission shows a tumoral lesion with a high degree of accumulation with a maximum standardized uptake value = 16.73

**Fig. 2:**
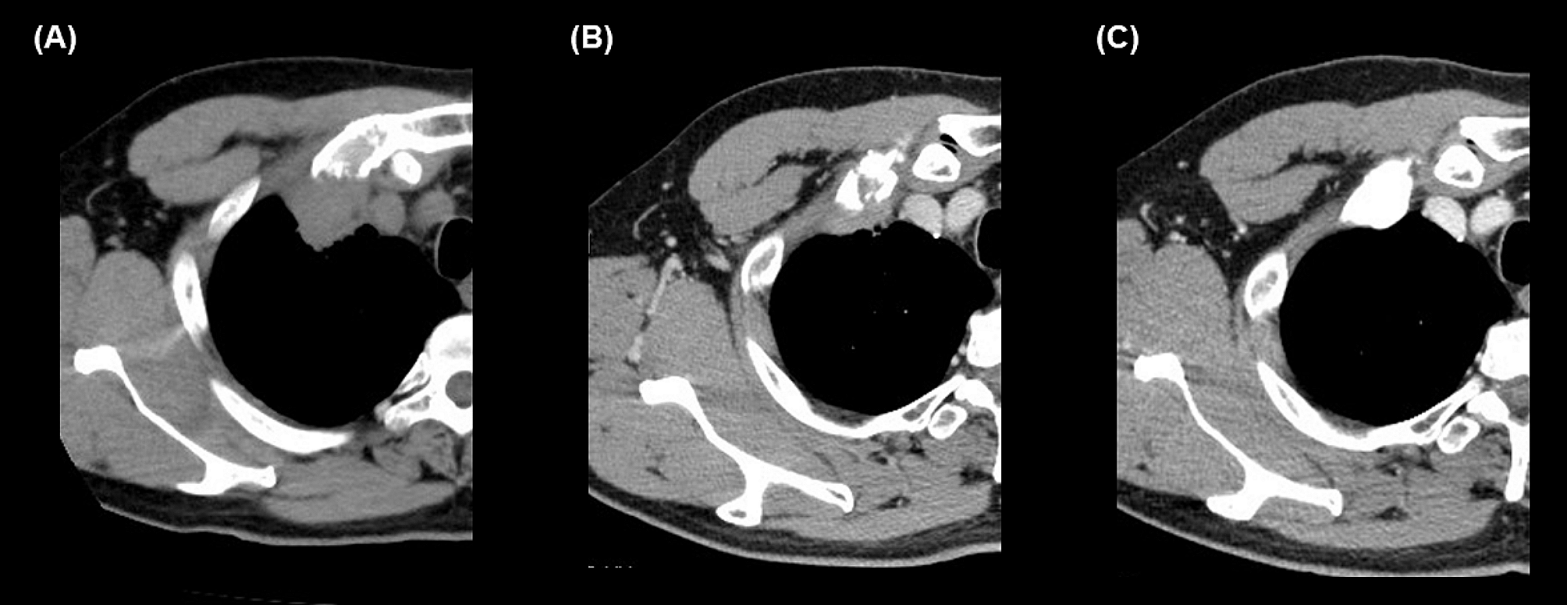
Clinical course. Computed tomography on the 12th day post-admission shows a tumoral lesion suspicious of infiltration into the right first rib and intercostal muscles **(A).** On the 47th day post-admission, computed tomography shows that the mass had shrunk and flattened since the previous 8 F-Fluorodeoxyglucose positron emission tomography-computed tomography **(B).** On the 176th day post-admission, computed tomography shows that the right lung S1 segment has a scar-like appearance **(C)**

A 49-year-old man was diagnosed with asthma controlled without medication. Two months before presentation, the patient had no abnormal results during a health check. However, he experienced chest pain and visited our hospital’s outpatient department. The patient had no relevant family history, did not drink or smoke, and had no allergies. The laboratory data were unremarkable. Chest radiography revealed a mass at the proximal end of the right clavicle. Computed tomography (CT) showed an approximately 36-mm tumoral lesion suspicious of infiltration into the right first rib and intercostal muscles, with changes in the density of the lung parenchyma around the mass, making it difficult to distinguish whether the mass was intrapulmonary or extrapulmonary (Figs. 1A and 2A). The findings suggested an extension of the lesion along the lymphatic vessels and stroma. The pain consistent with the same area was so severe that a nonsteroidal anti-inflammatory drug failed to control the pain, and opioids were administered. A bronchoscopy performed on the 21st day post-admission failed to reach a diagnosis, and a computed tomography (CT)-guided biopsy was performed on the 25th day post-admission.

**Fig. 3:**
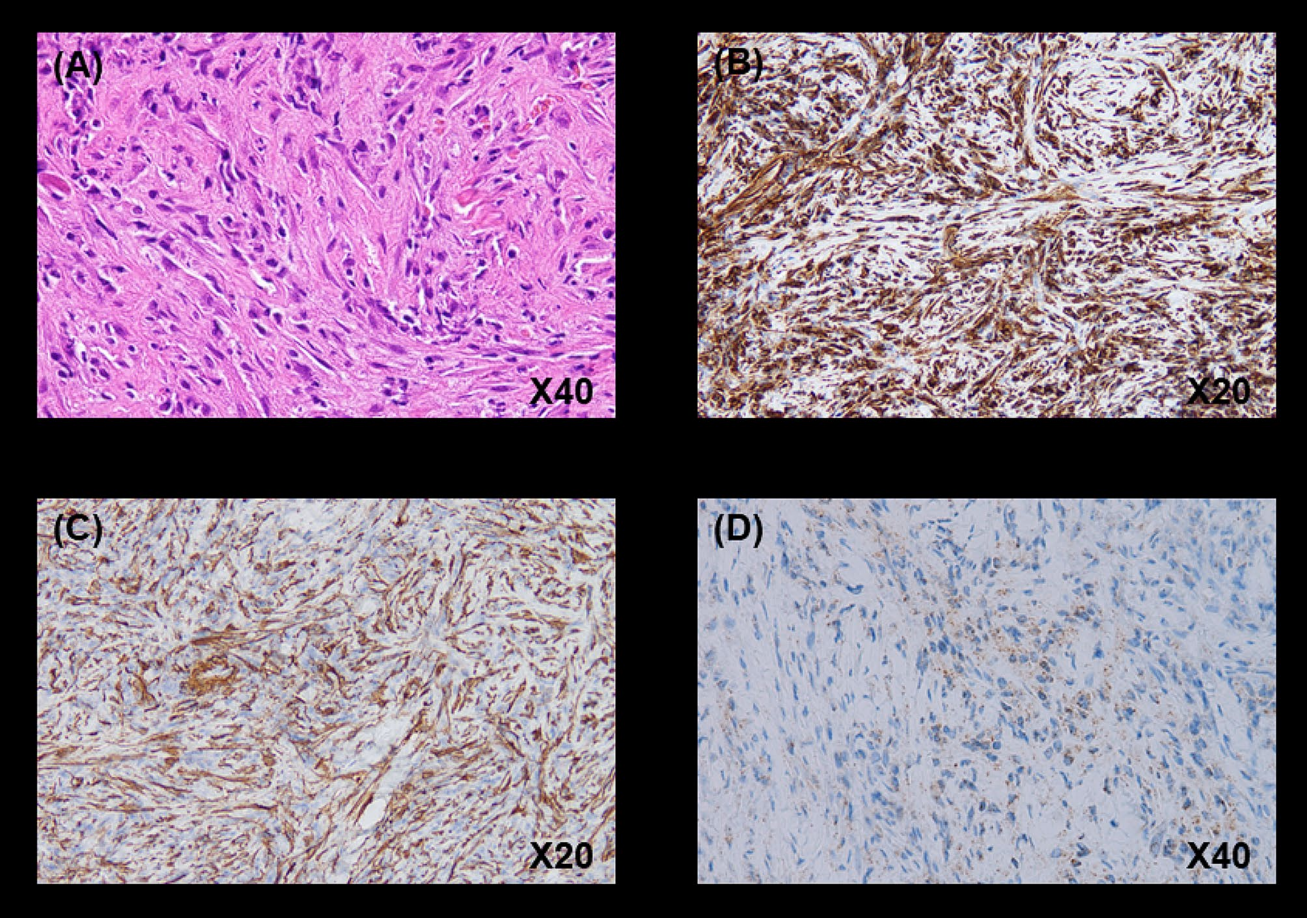
Photomicrographs of a computed tomography-guided percutaneous lung biopsy specimen. **(A)** Hematoxylin and eosin staining. The main component is collagenous fibrous tissue, which contains abundant inflammatory cells. Inflammatory cells are diverse, including neutrophils and lymphocytes. The fibrous tissue is partially vitiligo-like. In the areas with abundant fibroblast-like spindle-shaped cells, there is a mat-like appearance and no atypia. Immunostaining for vimentin **(B)** and α-smooth muscle actin **(C)** were positive, and immunostaining for Anaplastic lymphoma kinase with a D5F3 antibody reveals a faint positive image in half of the spindle-shaped cells **(D)**

Hematoxylin and eosin staining revealed a partially hyalinized collagenous fibrous tissue containing abundant inflammatory cells, including neutrophils and lymphocytes. In areas with abundant fibroblast-like spindle-shaped cells, storiform fibrosis, and no cellular atypia were observed (Fig. 3A).

Immunostaining was positive for vimentin (Fig. 3B) and α-smooth muscle actin (Fig. 3C) and negative for anti-cytokeratin (AE)1/AE3, cytokeratin 7, cluster of differentiation (CD) 34, desmin, soluble 100 protein (S-100), and B-cell lymphoma protein 2. CD3 and CD20 showed positive staining in background small lymphocytes, and the number of CD3-positive lymphocytes was higher than that of CD20-positive lymphocytes. CD138-positive plasma cells remained in nonspecific clusters. Ki67 positivity was approximately 5%. 18 F-fluorodeoxyglucose positron emission tomography/CT (18 F-FDG PET/CT) performed 4 days after CT-guided biopsy showed a tumoral lesion with a maximum standardized uptake value of 16.73 in the same area as the CT image, with no abnormal accumulation in other organs (Fig. 1B).

Based on these results, pathological malignant lymphoma was ruled out, and the patient was referred to the Respiratory Surgery Department in another hospital for surgical treatment. Additional immunostaining performed at the hospital showed that the samples were positive for nuclear BRCA1-associated protein-1 and methylthioadenosine phosphorylase; partially positive for D2-40; equivocal for protein HEG homolog 1; and negative for Calretinin, Epstein-Barr virus-encoded RNA (using in situ hybridization), Periodic acid–Schiff, Grocott, and Ziehl-Neelsen staining. No pathogens (Epstein–Barr virus-infected cells, fungi, or mycobacteria) were detected. Histopathological examination revealed that the tumor was predominantly fibroblastic, and there were no obvious malignant findings. Additional immunostaining for anaplastic lymphoma kinase (ALK) with a D5F3 antibody revealed a very faint positive image in half of the spindle-shaped cells (Fig. 3D).

Contrast-enhanced CT was scheduled as a preoperative examination; however, due to the patient’s pre-existing bronchial asthma, the decision was made to perform CT after administering steroids. Oral prednisolone (30 mg) was administered 12 and 2 h before contrast administration. The contrast-enhanced CT showed that the mass-like structure in S1b of the right lung had shrunk and flattened compared to the previous 18 F-FDG PET/CT findings. Further, the lesion appeared continuous from the lung parenchyma to the right first rib costochondral transition area (Fig. 2B), suggesting inflammatory changes and the presence of costochondritis. Thus, the lesion was suspected to be caused by an inflammatory change, such as costochondritis. The adjacent right first rib showed sclerotic changes and reactive changes suggestive of osteomyelitis or inflammation. The parenchymal side of the lung showed a discoid shadow along the bronchovascular side, indicating atelectasis.

Therefore, surgery was avoided, and CT imaging obtained 4 months later showed that the lesion in the right lung S1b subsegment had further shrunk and became scar-like (Fig. 2C).

After oral steroid administration, the tumor, which showed bone invasion, shrank in size. As for the cause of the shrinkage of the lesions, spontaneous remission was considered a possibility; however, it was suggested that the administration of corticosteroids may have been involved as a trigger.

Based on the pathological findings and disease course, the diagnosis of IMT was made. More than 1 year later, the patient has had no symptoms and no recurrence.

## Discussion and conclusions

Pathologically, IMTs show dense proliferation of various spindle-shaped cells (myofibroblasts) against a background of myxoedematous collagenous stroma and inflammatory cell infiltration, mainly plasma cells and lymphocytes [2]. Immunostaining has been reported to be positive for mesenchymal markers, such as vimentin (99%), smooth muscle actin (92%), and desmin (69%), but negative for cytokeratin, CD34, S-100, and epithelial membrane antigen [6]. Most reports suggest that surgical resection is the only effective treatment for IMT, while other studies report the efficacy of steroid therapy and nonsteroidal anti-inflammatory drugs. However, the latter has not yet been established as a standard treatment [1]. Carboplatin and Taxol; Bleomycin, Etoposide, and Cisplatin; vincristine/methotrexate; and Ifosfamide, Carboplatin, Etoposide, and Taxol regimens have been used in inoperable cases but have not been established as standard treatments [5, 7]. Reports of improvement with oral steroids alone, as shown in the present case, are rare. Conversely, the possibility that corticosteroids may promote IMT proliferation and relapse after steroid therapy has been reported [8, 9]. Although the response of IMTs to steroid therapy in combination with antibiotics is well known, some conclusions have not yet been reached [9]. The efficacy of combination therapy with radiation and glucocorticoids or methotrexate and glucocorticoids has been documented, but these case reports are rare [10, 11]. However, there are no reports of resolution of IMT with glucocorticoids alone or after a very short period of treatment. Moreover, some groups of patients with IMTs may be more susceptible to steroid therapy, and some may be more likely to experience subsequent relapses. However, since this is a rare disease, research has not been extensive. Several inducers have been hypothesized to increase the response to tissue injury in the formation of IMTs [2]. Previous case reports have demonstrated the involvement of local inflammation in the progression of IMTs, supporting a potential role for anti-inflammatory therapy. Adjuvant therapy with steroids, radiation therapy, chemotherapy, and nonsteroidal anti-inflammatory drugs has rarely been reported. Currently, there is no consensus regarding the most effective nonsurgical course of treatment [12].

Further, crizotinib use in an anaplastic lymphoma kinase-positive patient has been reported (not covered by insurance in Japan but approved by the US Food and Drug Administration in 2021) [13]. Gambacorti-Passerini et al. reported that almost two-thirds of objective responses to crizotinib in patients with IMTs persisted for more than 2 years, with a 67% progression-free survival at this time point. Despite the small number of patients enrolled, the findings support the National Comprehensive Cancer Network’s recommendation for crizotinib as a treatment for IMTs with ALK rearrangements and suggest that ALK testing should be performed routinely in all patients with IMTs [14]. In these patients, ALK expression detected by immunohistological staining can reach approximately 50% and correlates with gene rearrangement [2], which may be a reason for choosing crizotinib as the treatment option. Moreover, if the patient shows recurrence, additional immunostaining may expand treatment options.

Some cases of IMTs may metastasize or recur, and patients should be regularly monitored in anticipation of this possibility. If recurrence is observed after follow-up with glucocorticoid monotherapy, re-administration of glucocorticoids might be considered one of the effective treatments. However, since this disease has no established treatment, surgery should be considered if the lesions are resectable; if they are extensive, crizotinib or chemotherapy should be considered.

## Data Availability

All data and materials are available for sharing if needed.

## Abbreviations

IMT: Inflammatory myofibroblastic tumor
IL: Interleukin
CT: Computed tomography
AE: Anti-cytokeratin
CD: Cluster of differentiationS-100:Soluble 100 protein
18F-FDG PET/CT: 18 F-Fluorodeoxyglucose positron emission tomography-computed tomography
ALK: Anaplastic lymphoma kinase
